# Position-Specific Analysis and Prediction of Protein Pupylation Sites Based on Multiple Features

**DOI:** 10.1155/2013/109549

**Published:** 2013-08-26

**Authors:** Xiaowei Zhao, Jiangyan Dai, Qiao Ning, Zhiqiang Ma, Minghao Yin, Pingping Sun

**Affiliations:** ^1^College of Computer Science and Information Technology, Northeast Normal University, 2555 Jingyue Street, Changchun 130117, China; ^2^Key Laboratory of Intelligent Information Processing of Jilin Universities, Northeast Normal University, Changchun 130117, China

## Abstract

Pupylation is one of the most important posttranslational modifications of proteins; accurate identification of pupylation sites will facilitate the understanding of the molecular mechanism of pupylation. Besides the conventional experimental approaches, computational prediction of pupylation sites is much desirable for their convenience and fast speed. In this study, we developed a novel predictor to predict the pupylation sites. First, the maximum relevance minimum redundancy (mRMR) and incremental feature selection methods were made on five kinds of features to select the optimal feature set. Then the prediction model was built based on the optimal feature set with the assistant of the support vector machine algorithm. As a result, the overall jackknife success rate by the new predictor on a newly constructed benchmark dataset was 0.764, and the Mathews correlation coefficient was 0.522, indicating a good prediction. Feature analysis showed that all features types contributed to the prediction of protein pupylation sites. Further site-specific features analysis revealed that the features of sites surrounding the central lysine contributed more to the determination of pupylation sites than the other sites.

## 1. Introduction

As the firstly identified posttranslational small protein modifier in prokaryotes, prokaryotic ubiquitin-like protein (Pup) in* Mycobacterium tuberculosis* (Mtb) is an important signal for the selective degradation of proteins [1]. Pup attaches to substrate lysine via isopeptide bonds in a manner reminiscent of ubiquitin (Ub) and ubiquitin-like modifier (Ubl) conjugation to proteins in eukaryotes [2]. Although pupylation and ubiquitylation have functional similarity, the enzymology of pupylation and ubiquitylation is different [3]. Generally, there are three-steo reaction and three kinds of enzymes participating in the eukaryotic ubiquitylation process, including ubiquitin-activating enzymes, ubiquitin-conjugating enzymes, and ubiquitin ligases [4, 5], but only two-step reaction and two enzymes are participating in the prokaryotic pupylation process. Firstly, the Pup-GGQ C-terminal is deamidated to -GGE by deamidase of Pup [6], and then the proteasome accessory factor A (PafA) attaches the deamidated Pup to specific lysine residues of substrates [7].

Since identification of protein pupylation sites are of fundamental importance to understand the molecular mechanism of pupylation in biological systems, much interest has focused on this field, and large-scale proteomics technology has been applied to identify pupylation proteins and pupylation sites [8–10]. However, the experimental determination of exact modified sites of pupylated substrates is labor-intensive and time-consuming, especially for large-scale data sets. In this regard, the computation approaches which could effectively and accurately predict the pupylation sites is urgently needed. Liu et al. had constructed the first online predictor, GPS-PUP, for the prediction of the pupylation sites [11]. In their method, 127 experimentally identified pupylation sites in 109 prokaryotic proteins had been utilized as the training dataset, with an accuracy of 0.789 and a MCC of 0.286. 

In this study, a new predictor was developed to predict pupylation sites based on amino acid sequence features. Firstly, five kinds of features, which describe each amino acid of pupylation site and its surrounding ones, were extracted from each protein sequence, including physicochemical/biochemical properties of amino acids, Position-Specific Scoring Matrices (PSSM) which contain evolution information of amino acids, structural disorder of amino acids, second structure, and solvent accessibility. Secondly, the maximum relevance minimum redundancy (mRMR) and incremental feature selection methods were made on five kinds of features to find the optimal feature set. Finally, the predictor model was built based on the optimal feature set with the assistance of the support vector machine algorithm. For the new constructed pupylation sites dataset, the accuracy of the proposed predictor was 0.764 on the training dataset, and the MCC was 0.522. Compared with GPS-PUP, our predictor has the following features: (1) a larger benchmark dataset was used; (2) our study showed how much important the roles these features played in the prediction. Our feature analysis shows that evolutionary information and physicochemical/biochemical properties played important role in the recognition of pupylation sites, and sites 7, 10, and 11 contributed the most to the determination of pupylation sites. (A web server for predicting pupylation sites was developed and is available at http://210.47.24.217:8080/PrePup/).

## 2. Materials and Methods

### 2.1. Dataset

The pupylated proteins used in this study were extracted from PupDB [3]. Protein sequences with less than 50 amino acids were excluded because they may be just fragments [12, 13]. Protein sequences including nonstandard amino acids, such as “B,” “J,” “O,” “U,” “X,” and “Z,” were excluded as well. As a result, there were 182 pupylated proteins with 215 known pupylation sites. After a homology-reducing screening procedure by using CD-HIT [14, 15] to remove those proteins that had 40% sequence identity to any other, we finally got 153 pupylated proteins with 183 positive sites, which constructed the nonredundant training dataset in this study (see Supporting Information Text S1 available online at http://dx.doi.org/10.1155/2013/109549).

Subsequently, similar to the development of other PTM site predictors [16, 17], the sliding window strategy was utilized to extract positive and negative samples. After a preliminary evaluation, the optimal window size was 21 in this paper, with 10 residues located upstream and 10 residues located downstream the pupylation sites in the protein sequence. In order to ensure the peptides (sequence fragments) with a unified length, a nonexisting residue coded by “-” was used to fill the corresponding position. Peptides with pupylation lysine as the middle residue were positive samples, and the remaining peptides with nonpupylation lysine as the middle residue were negative samples. Since the numbers of pupylation lysine sites and the nonpupylation lysine sites were highly imbalanced, we randomly selected three times negative samples (non-pupylated lysine fragments) to match the positive ones (pupylated lysine fragments) in the training dataset.

### 2.2. Feature Construction

#### 2.2.1. Amino Acid Factors

 Amino Acid Index (AAIndex) [18, 19] database is a collection of numerical indices that stand for various physicochemical and biochemical properties of amino acids. Atchley et al. [20] did multivariable statistical analyses on AAIndex and produced five multidimensional and highly interpretable numeric patters of attributes: codon diversity, covariation reflecting polarity, molecular volume, secondary structure, and electrostatic charge. These five numerical pattern scores (called “amino acid factors”) have been used to successfully solve many biology problems [21–24]. Here, we also used these five amino acid factors to encode each amino acid of a given protein.

#### 2.2.2. PSSM Conservation Scores

 Evolutionary conservation always indicates important biology function, and posttranslational modifications are prone to occur in the conservation protein segments. In this study, we used Position Specific Iterated BLAST [25] (PSI-BLAST) to quantify the sequence conservation with Position-Specific Scoring Matrix (PSSM) which has been demonstrated to be effective for the identification of many posttranslational modification sites [26–30]. PSSM depicts the conservation of each amino acid in the sequence by a 20D numerical vector, each dimension of which measures the likelihood that the amino acid mutates to 20 different amino acids. The PSSM matrix for each of the proteins is generated by the “blastpgp” program of the PSI-BLAST package with three iterations of searching at cutoff *E*-value of 0.0001 for inclusion of sequences in subsequent iterations. And the alignment database is UniRef 100 (Release: 15.9).

#### 2.2.3. Structural Disorder Score

 Intrinsic disorder regions [31] are often rich in binding sites which are important loci for various protein posttranslational modifications such as methylation and phosphorylation [32]. Thus, we used the structural disorder feature of residue in the sequence to encode the peptides. VSL2 [33], which can accurately predict both long and short disordered regions in proteins, was utilized to calculate disorder score that represented the disorder status of each residue in a given protein sequence.

#### 2.2.4. Secondary Structure

 Protein structures play important roles in protein functioning and the posttranslational modification of specific residues may be influenced by the secondary structure of the relevant residues. Thus, we also used protein secondary structure to encode each peptide. In investigating secondary structures surrounding pupylation sites, PSIPRED [34] was utilized to predict the secondary structure from a given protein sequence. PSIPRED applied two feed-forward neural networks to predict the secondary structure using the results from PSI-BLAST. The result data of PSIPRED was encoded in terms of “C” for coil, “H” for helix, and “E” for strand. In order to transform these terms into numeric vectors, a 3D binary vector was used: coil (C) was encoded as “001,” helix (H) was encoded as “010,” and strand (E) was encoded as “100.”

#### 2.2.5. Solvent Accessibility

 It has been found that the posttranslational modifications of specific residues may be affected by the solvent accessibility [35]. Therefore, the solvent accessibility was also considered to encode each peptide. The SSPro program in the SCRATCH software package [36] was utilized to calculate the ASA value, which classified solvent accessibility of each amino acid as “buried” or “exposed,” encoded with “10” and “01,” respectively.

#### 2.2.6. The Feature Space

 Since the middle residues of the peptides were always the same and shared the common amino acid factors, these middle residues were thus encoded by 20 features of PSSM conservation scores, 1 feature of disorder score, 3 features of secondary structure, and 2 features of solvent accessibility, totally 26 features. Other residues were represented by 5 features of amino acid factors, 20 features of PSSM conservation scores, 1 feature of disorder score, 3 features of secondary structure, and 2 features of solvent accessibility, totally 31 features. Overall, each peptide consisting of 21 amino acid residues was represented by 20 × 31 + 26 = 646 features.

### 2.3. Model Constructing

 After the encoding of each peptide in the training dataset, we firstly used maximum relevance, minimum redundancy [37, 38] to prioritize the 646 features according to their importance. Then, based on the order of the sorted features, we obtained 646 feature sets. For each feature set, a prediction model was built with the nearest neighbor algorithm and evaluated by the jackknife cross-validation. The incremental feature selection method was then used to find the optimal feature set corresponding to the best prediction performance. Finally, the optimal feature set was input into support vector machine classifier to establish the final prediction model.

### 2.4. Prediction Algorithms

 In this study, nearest neighbor algorithm (NNA) was used to find the optimal feature subset. NNA predicts an unknown sample to share the common class as its nearest neighbor. For details on this algorithm, readers are advised to refer to [39].

Support vector machine (SVM) is a popular machine learning algorithm mainly used in dealing with binary classification problem. In this paper, LIBSVM package [40] with radial basis kernels (RBF) is used, where the kernel width parameter *γ* represents how the samples are transformed to a high-dimensional space. Grid search strategy based on 5-fold cross-validation is utilized to find the optimal parameters *C* and *γ* ∈ {2^−7^, 2^−6^,…, 2^8^}, so that a total number of 256 grids are evaluated.

### 2.5. Performance Assessment

The jackknife cross-validation test is adopted here [41, 42], since the outcome obtained by it is always unique for a given benchmark dataset, and has been widely used to examine the performance of various predictors [41, 43–45]. In the jackknife cross-validation process, the proteins are singled out from the dataset one by one as a testing protein, and the classifier is trained by the remaining proteins.

In order to evaluate the predictor proposed in this study, four measurements are used: sensitivity (Sn), specificity (Sp), accuracy (Ac) and Matthews correlation coefficient (MCC). For the definition of these four measurements, readers are advised to refer to [17]. In addition, the receiver operating characteristic (ROC) curves and the area under the curve (AUC) value are also carried out.

## 3. Results and Discussion

### 3.1. The Ordered Features by mRMR

 By running the mRMR software, we obtained two ranked feature lists (see Supporting Information Text S2): (1) the MaxRel feature list that contained all the 646 features ordered by their relevance to the class of samples, (2) the mRMR feature list that contained all the 646 features ordered by the maximum relevance and minimum redundancy criteria. Within these two lists, a smaller index of a feature meant that it was more important in discriminating pupylation sites from nonpupylation sites. The mRMR feature list was used in the following IFS procedure for the selection of the optimal feature set.

### 3.2. IFS Result and the Optimal Feature Set

**Figure 1:**
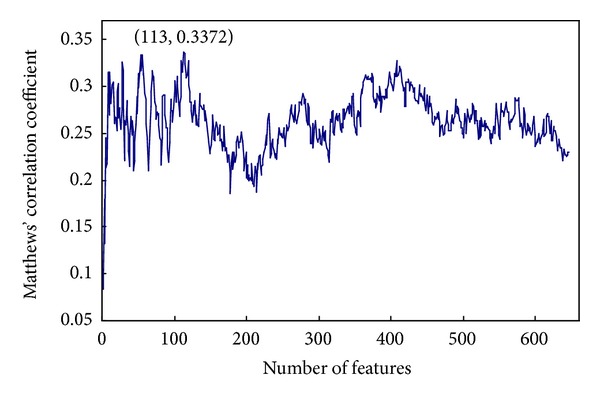
The IFS curve showed the values of MCC against feature numbers based on the data in Supporting Information Text S2. The maximum MCC was 0.3372 when 113 features were used. These 113 features were considered as the optimal feature set of our classifier.

 By adding the ordered features one by one, we constructed 646 feature sets. For each feature set, the predictor was built using the nearest neighbor algorithm and evaluated by the jackknife cross-validation. The IFS results can be seen in Supporting Information Text S3.  Figure 1 showed the IFS curve plotted based on the data in Supporting Information Text S2, and the curve reached its peak with the MCC of 0.337 and the number of features was 113. So, these 113 features (see Supporting Information Text S4) were regarded as the optimal feature set of our predictor. The predictive sensitivity, specificity, and accuracy based on these 113 features were 0.541, 0.792, and 0.709, respectively.

### 3.3. Biological Feature Analysis of the Optimal Feature Set

**Figure 2:**
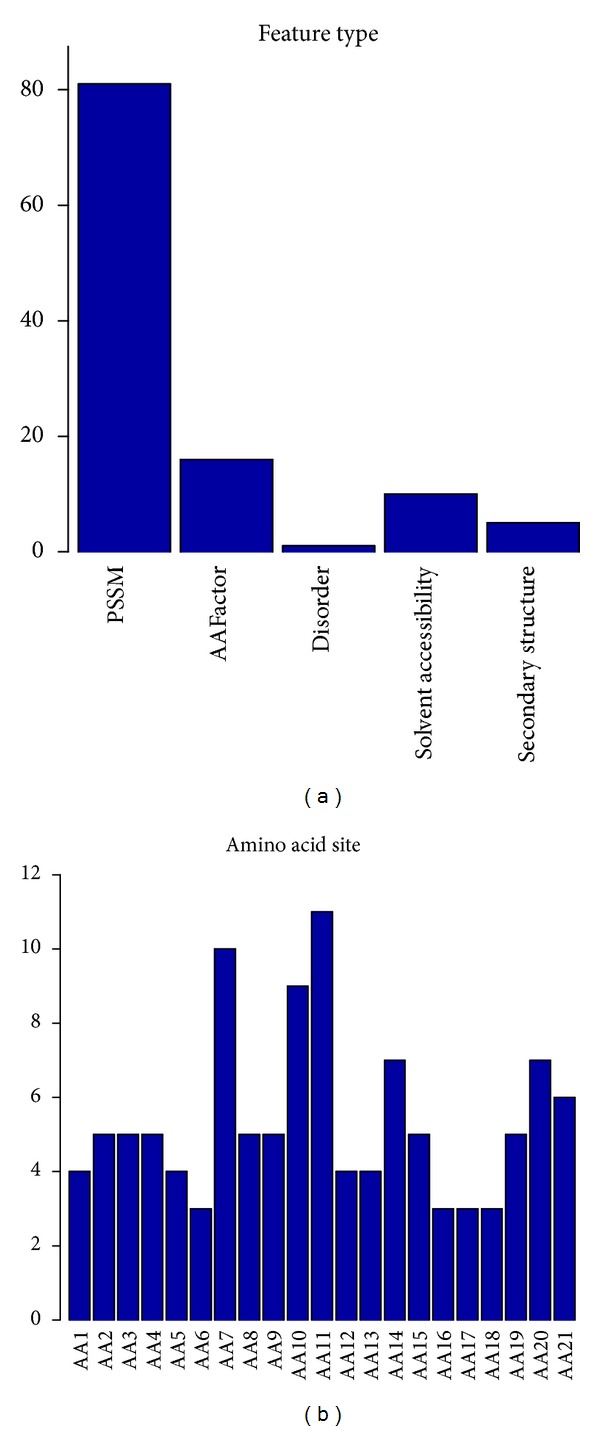
The number of each type or each site of features in the optimal feature set. (a) Feature distribution of the 113 optimal features. (b) Site specific distribution of the 113 optimal features.

 As described in Section 2, there were five kinds of features: amino acid factors, PSSM conservation scores, structural disorder scores, secondary structure, and solvent accessibility. The number of each type of features in the optimal feature set was investigated and shown in Figure 2(a). In the 113 optimal features, there were 81 features of PSSM conservation score, 16 features of amino acid factor, 1 feature of disorder, 10 features of solvent accessibility, and 5 features of secondary structure, indicating that all types of features played some roles in the determination of pupylation sites, and PSSM conservation score may play an irreplaceable role in pupylation sites prediction. The number of each site of features in the optimal feature set was shown in Figure 2(b). In can be clearly seen from Figure 2(b) that sites 7, 10, and 11 influenced mostly the determination of pupylation, and sites 14, 20, and 21 have a relatively small effect on pupylation, and sites 1–6, 8, 9, 12, 13, and 15–19 have the smallest effect on pupylation. The site-specific distribution of the 113 optimal features revealed that the residues at the left side of the pupylation site were more important for pupylation prediction than the other sites.

### 3.4. Biological Feature Analysis of the PSSM Conservation Score

**Figure 3:**
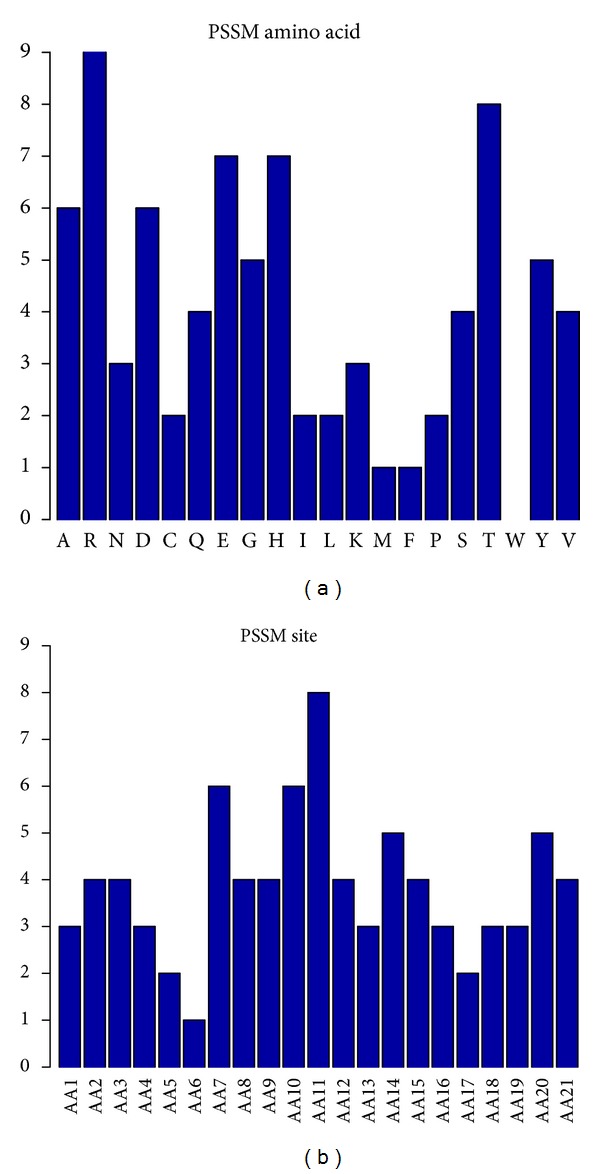
The number of each type or each site of PSSM features in the optimal feature set. (a) The number of each type of PSSM features in the optimal feature set. (b) The number of each site of PSSM features in the optimal feature set.

 As previously mentioned, there were 81 features of PSSM conservation score, which had the greatest proportion of the 113 optimal features. Therefore, we investigated the number of each type of PSSM features in the optimal feature set (see Figure 3(a)) and found that the conservation against mutations to different amino acids has different effect on the determination of pupylation sites. Mutations to amino acids R, T, E, and H have a larger influence on pupylation than mutations to other amino acids. The first feature in the mRMR feature list (Supporting Information Text S4) was the conservation status against residue K, which meant that the conservation of lysine was very important for predicting of pupylation sites. We also investigated the number of each site of PSSM features in the optimal feature set. It can be seen from Figure 3(b) that the conservation of lysine site (AA11) played the most important role in the determination of pupylation sites, and the conservation status of the sites 7, 10, 14, and 20 also played relatively more roles than the other sites. Particularly, the amino acid at site 6 has been shown to be imperfectly conserved and in most case was a D residue. There were seven PSSM features in the top 10 features of the optimal feature set: the conservation status against residue K at site 11, the conservation status against residue E at site 10, the conservation status against residue E at site 7, the conservation status against residue S at site 10, the conservation status against residue V at site 19, the conservation status against residue K at site 8, and the conservation status against residue E at site 14. This may suggest that conservation influenced more the pupylation sites prediction.

### 3.5. Biological Feature Analysis of the Amino Acid Factor

**Figure 4:**
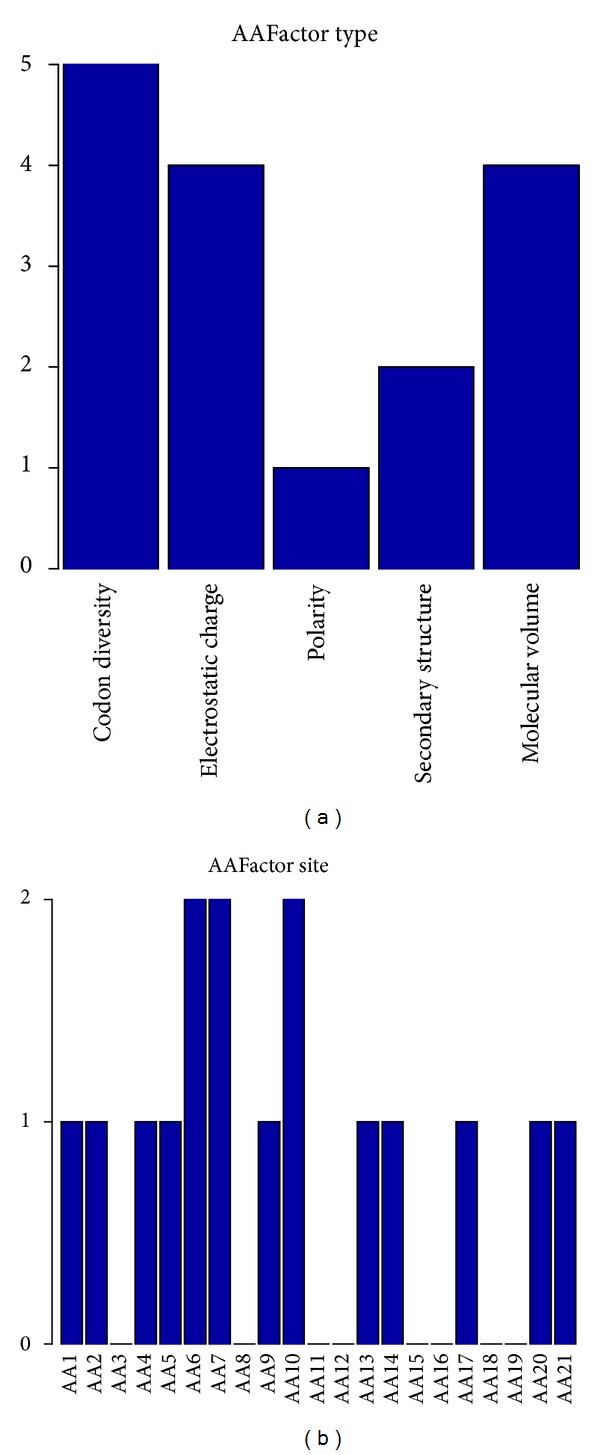
The number of each type or each site of amino acid factor features in the optimal feature set. (a) The number of five different types of amino acid factor features in the optimal feature set. (b) The number of each site of PSSM features in the optimal feature set.

Figure 4 showed the feature- and site-specific distribution of the amino acid factor features in the optimal feature set. It can be seen from Figure 4(a) that the codon diversity, electrostatic charge, and molecular volume were almost equally important features in the determination of pupylation sites. The polarity and secondary structure amino acid factor features have a small influence on pupylation sites prediction. In Figure 4(b), residues at sites 6, 7, and 10 have the most important effect in the determination of pupylation sites, and the other sites 1-2, 4-5, 9, 13-14, 17, and 20-21 were almost equally important. Among these sites, sites 6 and 7 were located in the upstream of the pupylation sites. The electrostatic charge of site 20 had an index of 6 in the optimal feature set, indicating that it was an important feature for the prediction of pupylation sites.

### 3.6. Biological Feature Analysis of the Solvent Accessibility

**Figure 5:**
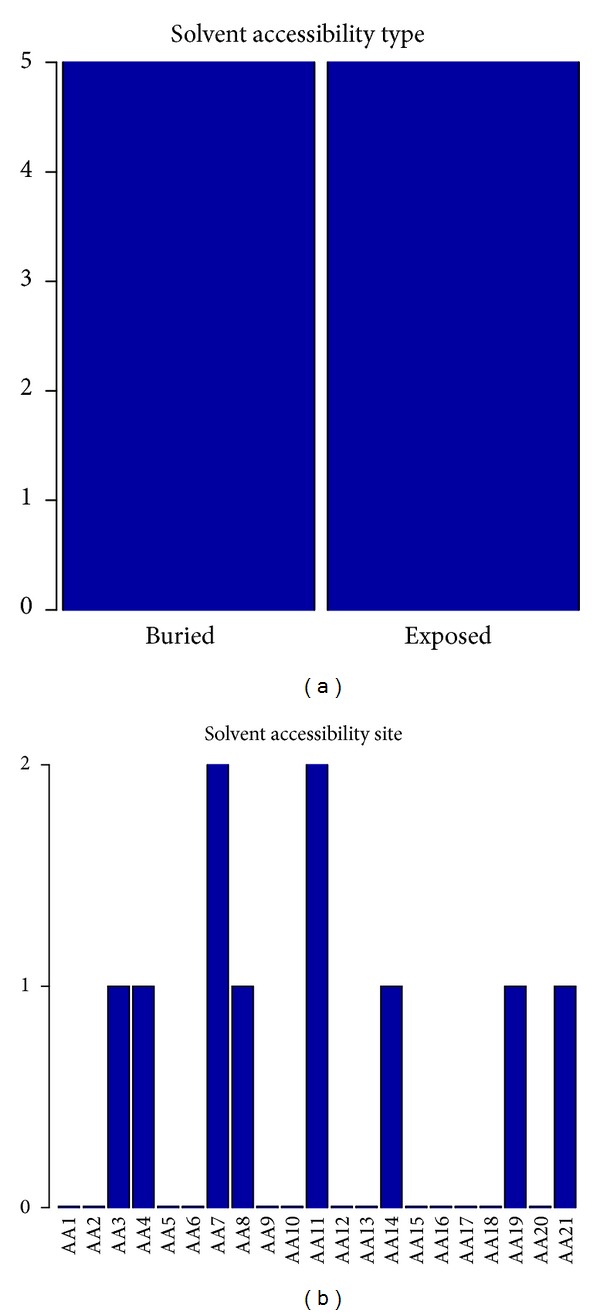
The number of each type or each site of solvent accessibility features in the optimal feature set. (a) The number of two types of solvent accessibility features (buried and exposed) in the optimal feature set. (b) The number of each site of solvent accessibility features in the optimal feature set.

 The number of each type of and the number of each site of solvent accessibility features in the optimal feature set has been investigated. It can be clearly seen from Figure 5(a) that the number of two types of solvent accessibility (buried and exposed) was equal. That is to say, both types of solvent accessibility features had equal impact on the determination of pupylation sites. Moreover, as can be seen from Figure 5(b), residues at sites 7 and 11 played the most important roles in the determination of pupylation sites than the other sites. There were 2 solvent accessibility features in the top 10 features: the solvent accessibility feature of site 11 had an index of 3, and the solvent accessibility feature of site 21 had an index of 4.

### 3.7. Biological Feature Analysis of the Disorder Score

 In the optimal feature set, there was only 1 disorder feature. A reasonable explanation was that the nearby residue's disorder statue had an important influence on pupylation modification process. This disorder feature of site 10 had an index of 72.

### 3.8. Biological Feature Analysis of the Secondary Structure

**Figure 6:**
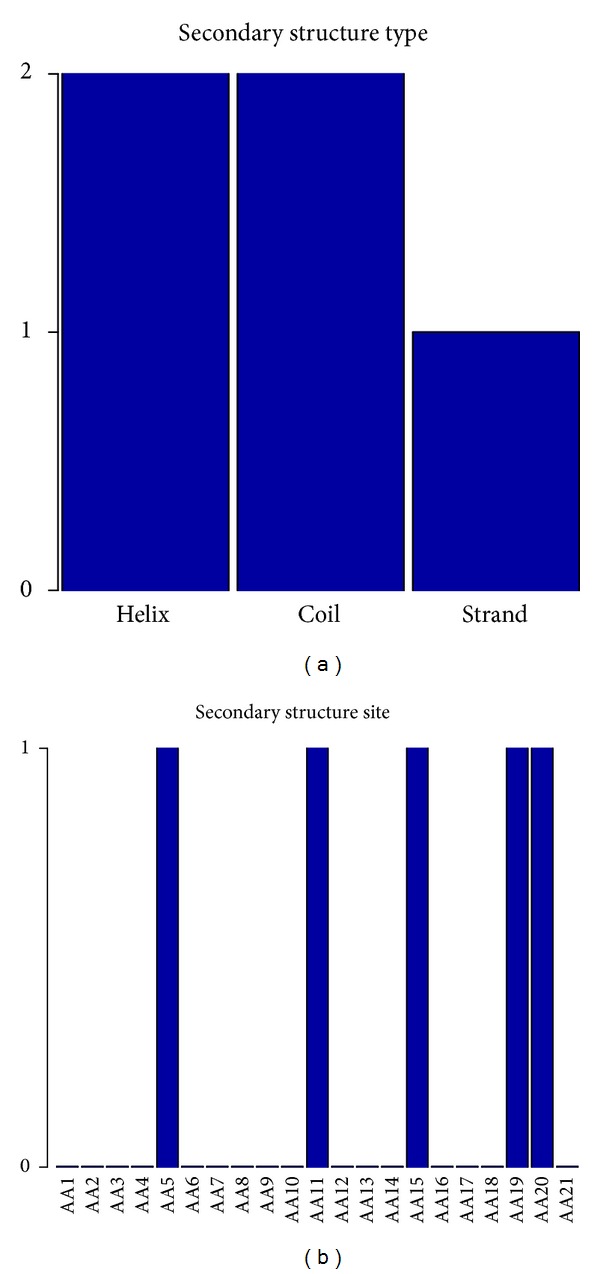
The number of each type or each site of secondary structure features in the optimal feature set. (a) The number of three types of secondary structure features (helix, coil and strand) in the optimal feature set. (b) The number of each site of secondary structure features in the optimal feature set.

 The feature- and site-specific distribution of the secondary structure features in the optimal feature set was shown in Figure 6. The number of the three types of 5 secondary structure features (helix, coil, and strand) in the optimal feature set was investigated and shown in Figure 6(a), from which we can see that all types of secondary structure features affected the pupylation sites prediction. Moreover, in Figure 6(b), residues at the sites 5, 11, 15, 19, and 20 have relatively more impact on the determination of pupylation sites.

### 3.9. Comparisons with Other Methods

**Figure 7:**
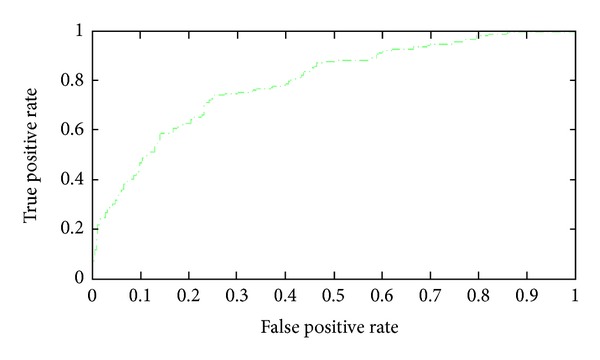
ROC curves of the SVM-based method for pupylation sites prediction; the AUC value was 0.791.

 When the 113 optimal features were input into the NNA classifier, the predictive sensitivity, specificity, accuracy, and MCC were 0.541, 0.792, 0.709, and 0.337, respectively. We also put the 113 optimal features into a SVM classifier, and the predictive sensitivity, specificity, accuracy, and MCC were 0.522, 0.938, 0.764, and 0.522, respectively. The ROC curve of the SVM classifier was given in Figure 7, and the AUC value was 0.791. Overall, the SVM-based method was better than the NNA-based method for pupylation sites prediction, and we adopted this model as our final prediction model. 

We have demonstrated that the proposed method could achieve a promising prediction performance for pupylation sites prediction. To objectively evaluate our proposed predictor, we further compared the proposed predictor with GPS-PUP [11]. Liu et al. searched PubMed with the keywords of “pupylation” and “prokaryotic ubiquitin” and collected 127 experimentally identified pupylation sites in 109 prokaryotic proteins. Since we did not know the ratio of positive to negative samples in their training dataset, we established a prediction model based on a training dataset in which the negative samples were three times the positive ones and only reported the sensitivity of the prediction model. The sensitivity of our method was 0.739, and the sensitivity of GPS-PUP was 0.448 when the threshold was medium. That is to say, the sensitivity of our proposed method was better than that of GPS-PUP.

### 3.10. Direction for Experimental Validation

 By means of the mRMR feature selection method, an optimal feature set including 113 features was selected. We analyzed the feature- and site-specific distribution of each kind of features in the optimal feature set. As a result, we found that evolutionary information and physicochemical/biochemical properties played an important role in the recognition of pupylation sites. Sites 7, 10, and 11 contributed the most to the determination of pupylation sites. Particularly, the residues located in the upstream of the pupylation sites may play an important role in pupylation modification process. The selected features at different sites could provide some useful clues for understanding the mechanism of pupylation process and guide experimental validation.

## 4. Conclusion

 In this study, a wide range of features had been combined to predict pupylation sites, including physicochemical/biochemical properties of amino acids, Position-Specific Scoring Matrices (PSSM) which contain evolution information of amino acids, structural disorder of amino acids, second structure, and solvent accessibility. Unlike other reports, we not only improved the prediction performance, but also analyzed how much important the roles these features played in the prediction. With the selected optimal feature set, our predictor reached a sensitivity of 0.522, a specificity of 0.937, and an accuracy of 0.764. Although the results obtained here were very promising, further investigation was needed to further clarify the mechanism of pupylation process.

## Supplementary Material

The supplementary material includes the non-redundant training dataset of our manuscript.
